# Plant Phytochromes and their Phosphorylation

**DOI:** 10.3390/ijms20143450

**Published:** 2019-07-13

**Authors:** Quyen T. N. Hoang, Yun-Jeong Han, Jeong-Il Kim

**Affiliations:** Department of Biotechnology and Kumho Life Science Laboratory, Chonnam National University, Gwangju 61186, Korea

**Keywords:** plant photoreceptors, protein kinase, autophosphorylation, reversible phosphorylation, light signaling

## Abstract

Extensive research over several decades in plant light signaling mediated by photoreceptors has identified the molecular mechanisms for how phytochromes regulate photomorphogenic development, which includes degradation of phytochrome-interacting factors (PIFs) and inactivation of COP1-SPA complexes with the accumulation of master transcription factors for photomorphogenesis, such as HY5. However, the initial biochemical mechanism for the function of phytochromes has not been fully elucidated. Plant phytochromes have long been known as phosphoproteins, and a few protein phosphatases that directly interact with and dephosphorylate phytochromes have been identified. However, there is no report thus far of a protein kinase that acts on phytochromes. On the other hand, plant phytochromes have been suggested as autophosphorylating serine/threonine protein kinases, proposing that the kinase activity might be important for their functions. Indeed, the autophosphorylation of phytochromes has been reported to play an important role in the regulation of plant light signaling. More recently, evidence that phytochromes function as protein kinases in plant light signaling has been provided using phytochrome mutants displaying reduced kinase activities. In this review, we highlight recent advances in the reversible phosphorylation of phytochromes and their functions as protein kinases in plant light signaling.

## 1. Introduction

As sessile organisms, plants utilize light not only as the energy source for photosynthesis but also as environmental cues for photomorphogenesis, i.e., growth and development in response to light signals such as wavelength, intensity, direction, and duration [1,2]. Plants need to constantly monitor environmental light changes to optimize their growth and development, which makes plants evolve multiple photoreceptor systems including red (R)/far-red (FR) light-absorbing phytochromes [3,4]. Phytochromes are dimeric chromoproteins with each monomer possessing a covalently linked open tetrapyrrole phytochromobilin as a chromophore, which exist in spectrally distinct R light-absorbing Pr and FR light-absorbing Pfr forms [5]. In higher plants, phytochromes are encoded by small gene families, for example, five members (phyA to phyE) in *Arabidopsis thaliana* [6]. They are further classified into light-labile type I (phyA) and light-stable type II (phyB to phyE) species, among which phyA is responsible for sensing FR light and phyB to phyE play roles in R light-mediated photomorphogenic development [7,8]. In addition to protein stability, phytochromes exhibit the property of dark reversion, i.e., reversion of the active Pfr form into the inactive Pr form in the dark. As the most important type II phytochrome, phyB shows much faster dark reversion than phyA, which has recently been suggested to be an important characteristic to function as a thermosensor in plants [9,10].

Unlike animal photoreceptors that exist in highly specialized organs, plant phytochromes are ubiquitously present in all the tissues including roots [11]. Thus, phytochromes regulate almost every step of the plant life cycle, mediating various photomorphogenic processes, such as seed germination, de-etiolation, stem growth, pigmentation, and flowering [12]. Especially, plant growth between seed germination and the emergence of the first true leaves is pivotal for survival [13]. When germinated in the dark, plant seedlings undergo etiolated growth, i.e., skotomorphogenesis that is characterized by elongated hypocotyls and closed cotyledons with apical hooks. This etiolated development allows the buried seeds to emerge through the soil in search of light. In contrast, the de-etiolated development upon exposure to light (i.e., photomorphogenesis) is characterized by inhibition of hypocotyl elongation, hook opening, cotyledon expansion, and greening to allow seedlings for the autotrophic growth. Thus, the ability to switch from skotomorphogenesis to photomorphogenesis is essential for the successful establishment of seedlings, which is mediated by plant photoreceptors including phytochromes.

During skotomorphogenesis, phytochromes are biosynthesized as the inactive Pr form in the cytoplasm, which can be phototransformed into the active Pfr form upon exposure to R light. The Pr-to-Pfr phototransformation makes phytochromes to be localized into the nucleus where they interact with downstream signaling components and induce a highly regulated signaling network for the transcription of photoresponsive genes in plants [14,15]. A previous genome-wide expression analysis has shown that approximately 10% of the genes in the *A. thaliana* genome (~2500 genes) are regulated by phytochromes under prolonged exposure to light, in which about 250 genes are affected at least two-fold by continuous R light within one hour [16]. Approximately 80% of the light-responding genes are induced, and about 20% repressed. The majority of these genes are strongly related to the photomorphogenic changes to transit from heterotrophic to autotrophic growth, which include photosynthetic, hormone pathway-related and metabolic genes. Thus, the principal regulatory mechanism of phytochromes for photomorphogenesis might be the transcriptional regulation of photoresponsive genes via negative transcriptional factors such as phytochrome-interacting factors (PIFs) and positive transcriptional factors such as HY5 (elongated hypocotyl 5) and HYL (HY5-like) [17,18].

In addition to photomorphogenic development, photochromes are also involved in other physiological responses such as abiotic stress tolerance, plant defense, stomatal opening, and photosynthetic processes [19,20,21,22]. However, the involvement of these physiological responses is largely dependent on the transcription regulation of PIFs and HY5 by phytochromes, because it has been demonstrated that these transcriptional factors are important for the regulation of various physiological processes such as hormone, nutrient, abiotic stress (salt, drought, cold, elevated temperature), biotic stress, and reactive oxygen species signaling pathways, as well as photomorphogenic and photosynthetic processes [23,24]. In addition, phytochromes are also known to interact with other photoreceptors such as phototropins for the regulation of phototropism and stomatal opening [25,26]. Thus, phytochromes play multifaceted roles in plant growth and development, so this review cannot cover all of these aspects of phytochromes. Rather, we focus on the molecular mechanisms of phytochromes for the photomorphogenic development. Although phytochromes have been discovered several decades ago and significant advances have been made in establishing the phytochrome-mediated light signaling pathways, the initial biochemical and molecular mechanisms for phytochrome function have not been fully elucidated. In this review, we highlight current knowledge about the reversible phosphorylation of phytochromes and their functions as protein kinases in plant light signaling.

## 2. Domain Structure of Plant Phytochromes

**Figure 1:**
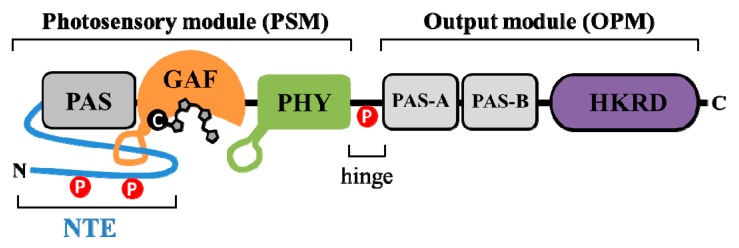
A representative domain structure of plant phytochromes. The N- and C-termini of protein are indicated, and a chromophore is covalently attached to a cysteine residue in the cGMP phosphodiesterase/adenylyl cyclase/FhlA (GAF) domain. The photosensory module (PSM) consists of N-terminal extension (NTE) and the photosensory core (PAS/GAF/PHY), and the output module (OPM) contain PAS-related domain (PRD) with a pair of PAS repeats (labeled as PAS-A and PAS-B) and histidine kinase-related domain (HKRD). The PSM and OPM are linked with each other by a hinge region, and the knot lasso motif in GAF domain and the hairpin motif in the phytochrome-specific (PHY) domain are indicated by the orange and green loops, respectively. The phosphorylation sites of plant phytochromes have been reported in the NTE and hinge regions (see below).

The phytochrome molecule consists of a globular N-terminal photosensory module (~65 kDa) and a structurally extended C-terminal output module (~55 kDa) [27]. The two domains are connected via a flexible hinge region (Figure 1). A previous domain swap experiment demonstrated that the N-terminal photosensory modules of phytochromes determine their photosensory specificity and differential light lability [28]. The photosensory module (PSM) consists of N-terminal extension (NTE), Per/Arnt/Sim (PAS), cGMP phosphodiesterase/adenylyl cyclase/FhlA (GAF), and a phytochrome-specific (PHY) domain. The phytochromobilin chromophore is covalently attached to a conserved cysteine residue in the GAF domain through a thioether linkage [29]. The PAS-GAF-PHY tri-domain is also known as the photosensory core that is enough to absorb light and to induce conformational changes [30]. The NTE is dispensable for the chromophore binding but necessary for the biological activity [31]. The PAS and GAF domains are necessary for the bilin lyase activity to attach the chromophore and the PHY domain is necessary to stabilize the Pfr form and to transmit the light signal to the C-terminal output module [32,33]. Only recently, a crystal structure of the PSM from a plant phyB was obtained, which showed an unusual figure-of-eight knot, called a “light-sensing knot lasso”, at the PAS-GAF interface and a well-ordered hairpin, also known as “tongue”, protruding from the PHY domain toward the chromophore pocket [34,35]. The light-sensing knot lasso motif is known to make a stable PAS-GAF interaction during Pr-to-Pfr photoconversion and also to serve as the binding motif with PIFs [36]. The tongue hairpin motif is suggested to be necessary for a shift of the PHY domain during the photoconversion, which mediates the association with the C-terminal output module [37].

The output module (OPM) consists of PAS-related domain (PRD) with a pair of PAS repeats (PAS-A and PAS-B) and histidine kinase-related domain (HKRD). The importance of OPM is previously highlighted by numerous missense mutations affecting this part of the protein [38,39]. The OPM of phytochromes has been reported to play roles in dimerization [40,41,42], nuclear localization [43,44], and signaling via protein-protein interaction with downstream components [14,45]. It has been suggested that the HKRD is a non-functional kinase domain due to the absence of key conserved residues within the histidine kinase domain [46]. Moreover, the N-terminal domain of phyB has been reported to be functional in plants when it can exist as dimers and be localized in the nucleus [47,48]. These results suggest that the C-terminal OPM is mainly necessary for dimerization and nuclear localization. Indeed, it has been recently reported that the HKRD functions as a dimerization domain for homo- and heterodimerization of phyB [42]. However, phyA requires the OPM for its full biological activity in plants, suggesting a signaling function of the domain. It is also notable that a few phytochrome-interacting proteins have been isolated by yeast two-hybrid screening using the HKRD as a bait, which includes PKS1 (phytochrome kinase substrate 1) and PAPP5 (phytochrome-associated protein phosphatase 5) [49,50]. Therefore, the OPM is likely to be important for the signaling of phytochromes, as well as their dimerization and nuclear localization. In addition, it should be noted that the functional roles of the OPM might be different between phyA and phyB.

## 3. Phytochrome-Mediated Light Signaling in Plants

Among research to elucidate the phytochrome-mediated light signaling pathway, an important paradigm shift occurred when experimental evidence for nuclear localization of phyB was provided [51]. Previously, most studies were focused on cytoplasmic events because it was widely accepted that phytochromes were cytoplasmic photoreceptors. Now, it is apparent that phytochromes accumulate in the nucleus upon photoactivation (i.e., Pr-to-Pfr transformation), clearly indicating that this is a crucial control step in the phytochrome signaling [52]. Following the nuclear translocation from cytoplasm, phytochrome responses are largely associated with massive changes in gene expression [16]. However, some phenomena cannot be explained through the transcriptional regulation of genes, because certain phytochrome responses are too fast to explain by a transcription-based action mechanism. For example, ion influx and calcium level changes were induced by brief exposures to R light in the etiolated oat coleoptiles [53]. Moreover, cytoplasmic functions of phyA was suggested from the analysis of Arabidopsis *fhl/fhy1* mutant; FHY1 (far-red elongated hypocotyl 1) and FHL (FHY1-like) are transport facilitators for phyA into the nucleus [54,55]. This study suggests the R-enhanced phototropism, abrogation of gravitropism, and the inhibition of hypocotyl elongation in blue light as phyA-specific cytoplasmic responses. Moreover, phyA was shown to interact with phototropins at the plasma membrane, and it is known that the phytochrome activity can influence plasma membrane H^+^-ATPase in guard cells for stomatal opening under R light [21,26]. These results suggest the functional roles of phytochromes in the cytoplasm and possibly in the plasma membrane. However, the molecular mechanisms for these phytochrome functions are not studied well. Moreover, it has been also suggested that most phyB responses require nuclear phyB, but not cytoplasmic phyB [47,56]. Thus, the phytochrome functions in the nucleus are considered as the main action mechanism, so this review focuses on the molecular mechanisms of phytochromes in the nucleus, especially in the aspects of phytochrome phosphorylation and their kinase activity.

**Figure 2:**
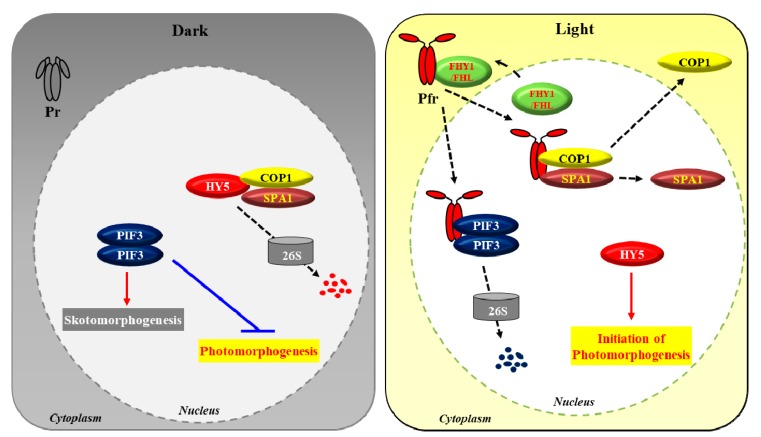
A simplified view of the phytochrome-mediated photomorphogenesis in *A. thaliana*. For simplicity, we use PIF3 as a representative of PIFs, HY5 as a representative of the master transcription factors of photomorphogenesis, and SPA1 as a representative of the SPA proteins. In the dark (left panel), phytochromes are biosynthesized as the inactive Pr forms, staying in the cytoplasm. Meanwhile, PIF3 proteins accumulate in the nucleus and regulate the expression of genes to prevent photomorphogenesis (shown as a blue T bar) by promoting skotomorphogenesis (shown as a red arrow). In addition, the COP1-SPA1 complex constantly degrade the expressed HY5 proteins via the ubiquitin 26S proteasome pathway to prevent photomorphogenesis (shown as a dotted black arrow). In the light (right panel), the photoactivated Pfr forms of phytochromes translocate into the nucleus (in the case of phyA, FHY1 and FHL are the facilitators for the import), where they can interact with downstream signaling components. They inactivate PIF3 by inducing protein degradation and the COP1-SPA1 complex by inducing its dissociation and subsequent nuclear exclusion of COP1, which all contribute to the accumulation of the master transcription factors for photomorphogenesis, such as HY5. Finally, HY5 induces the expression of light-responsive genes for photomorphogenic development. Movements of proteins are also shown as dotted arrows.

Before going into the reversible phosphorylation of phytochromes and their functions as protein kinases, it would be better to summarize the important steps for the phytochrome-mediated light signaling pathway in plants. Phytochromes mediate photomorphogenic responses from light input to physiological output such as seed germination and de-etiolation. Based on recent advances in understanding phytochrome signaling, a simplified view of the phytochrome-mediated light signaling pathway can be presented (Figure 2). In general, the phytochrome-mediated light signaling pathway is constructed as follows: photoactivation, nuclear localization, interaction with downstream signaling components, signal integration (for example, HY5 accumulation), and transcriptional control of photoresponsive genes [4,57]. 

The first step to initiate the phytochrome signaling is the conformational change between Pr and Pfr forms by sensing FR light by phyA and/or R light by phyB to phyE [5,58]. After the photoactivation, phytochromes translocate from the cytoplasm to the nucleus [15,59]. It is proposed that phytochromes might not possess an active endogenous nuclear localization signal (NLS), so the NLS-containing signaling partners are necessary for the light-induced translocation [60]. Although the transport facilitators for the import of phyB to phyE into the nucleus remain elusive, those for phyA have been identified as FHY1 and FHL [54,61]. FHY1 and FHL have a conserved C-terminal domain and the NLS and nuclear export signal (NES) sequences in the N-terminal domain. They interact directly with photoactivated phyA in the cytoplasm through their conserved C-terminal domain, and the phyA-FHY1/FHL complex is imported into the nucleus [62]. More recently, it is reported that the NLS of FHY1 is recognized by importin α (IMPα) independently of phyA, and phosphorylation on serine residues close to the NLS prevents FHY1 binding to IMPα [44]. In the nucleus, the photoactivated phytochromes interact with various downstream signaling components [45]. Among them, two components are critical to understanding the phytochrome signaling: a small set of phytochrome-interacting factors (PIFs) and the COP1 (constitutively photomorphogenic 1)-SPA (suppressor of *phyA-105*) complexes. 

PIFs are basic helix-loop-helix (bHLH) transcription factors that are known to be central players in phytochrome-mediated signaling networks [63]. They act to repress seedling photomorphogenesis including seed germination and de-etiolation while promoting seedling skotomorphogenesis through the regulated expression of more than a thousand genes [64,65]. Photoactivated phytochromes interact with PIFs in the nucleus, which induces phosphorylation of PIFs and subsequent protein degradation via the ubiquitin 26S proteasome proteolytic pathway [66,67,68]. Thus, it is established that phytochromes mediate photomorphogenic development by degrading PIFs, the negative regulators of photomorphogenesis. COP1 is a highly conserved E3 ubiquitin ligase from plants to animals and acts as a central repressor of photomorphogenesis in plants [69,70]. In addition, COP1 interacts with SPA family members (SPA1 to SPA4) that can enhance COP1 activity [71]. The COP1-SPA complexes are known to target multiple master transcription factors of photomorphogenesis for rapid degradation, which include HY5, LAF1 (long after far-red light 1), and HFR1 (long hypocotyl in far-red 1). Especially, HY5, a pivotal positive regulator of photomorphogenic development, is a basic leucine zipper (bZIP) transcription factor that binds directly to the promoters of light-inducible genes, promoting their expression and photomorphogenic development [24]. Photoactivated phytochromes have been shown to interact with COP1-SPA complexes, which induces the dissociation of the complexes [72,73]. Therefore, phytochromes negatively regulate COP1-SPA complexes to promote photomorphogenic development.

As shown in Figure 2, the main function of phytochromes would be the removal of negative regulators for photomorphogenesis. In the dark-grown seedlings, PIFs and COP1-SPA complexes function as the negative regulators to repress the initiation of photomorphogenesis. Rather, they promote and maintain etiolated development. In this condition, phytochromes exist in the cytoplasm as the inactive Pr forms, so there is no chance to interact with PIFs and COP1-SPA complexes that are located in the nucleus. In the light-grown seedlings, the photoactivated Pfr forms are translocated into the nucleus and can interact with PIFs and COP1-SPA complexes. The results of interactions are the inactivation of PIFs and COP1-SPA complexes by protein degradation and dissociation of the complex, respectively, which eventually induces the accumulation of HY5, i.e., one of the master transcriptional factors for photomorphogenesis. As a result, seedlings progress toward photomorphogenic development. Therefore, the photoactivated forms of phytochromes (Pfr) initiate the signals for photomorphogenesis by suppressing the functions of PIFs and COP1-SPA complexes. However, the biochemical and molecular mechanisms on how phytochromes induce the degradation of PIFs and the dissociation of COP1-SPA complexes are not elucidated well (see below).

## 4. Phosphorylation and Dephosphorylation of Plant Phytochromes

Reversible phosphorylation of a protein often serves as a signal modulation mechanism in the regulation of cellular activities. Plant phytochromes have been known as phosphoproteins by a phosphate analysis with immunoaffinity-purified proteins from dark-grown *Avena sativa* (oat) seedlings, indicating the presence of one mole of phosphate per mole of phytochrome monomer [74]. Later, the sites of phosphorylation have been identified using purified *A. sativa* phyA (AsphyA), and they are Ser-8 and Ser-18 in the NTE and Ser-599 in the hinge region (see Figure 1) [75]. Subsequently, functional analysis of the phosphorylation on the sites was performed. One important discovery was that phosphorylation at Ser-599 in the hinge region prevents the interaction of AsphyA with its downstream signaling components such as NDPK2 (nucleoside diphosphate kinase 2) [76]. These data suggest that the hinge region serves as a phosphorylatable signal-modulating site for the regulation of protein-protein interactions between phytochromes and downstream signaling partners. More recently, three sites (Ser-590, Thr-593, and Ser-602) in the hinge region of *Arabidopsis thaliana* phyA (AtphyA) are reported to be phosphorylated in plants, demonstrating that the hinge region plays an important role in regulating phosphorylation and function of AtphyA [77]. It is also notable that the phosphorylated form of AtphyA is produced in the nucleus by the Pfr form, suggesting the role of phytochrome phosphorylation in the nucleus.

Ser-8 and Ser-18 in the NTE have been identified as autophosphorylation sites of AsphyA and the autophosphorylation has been shown to provide a molecular mechanism for signal attenuation in the phyA-mediated light signaling pathway by accelerating protein degradation [78,79]. These data are consistent with previous results that Ser-to-Ala mutations or deletion of serines in the NTE region increased protein stability and biological activity of phyA [80,81,82,83]. Collectively, these data suggest that the autophosphorylation of these serine residues is involved in the desensitization of phyA signaling via accelerated protein degradation. In the case of phyB, Ser-86 in the NTE has been identified as the phosphorylation site in *A. thaliana*, and this phosphorylation inhibits phyB signaling via accelerated dark reversion [84]. These data suggest the different regulation of phyA and phyB by phosphorylation in the NTE region; phyA phosphorylation accelerates protein degradation, whereas phyB phosphorylation accelerates dark reversion (i.e., light-independent thermal conversion of Pfr to Pr). These differences might be due to the characteristics of phytochrome molecules. It is well-known that phyA is light-labile and mediates very low fluence response (VLFR) and FR-high irradiance response (FR-HIR) of plants, both of which do not show photoreversibility. Thus, phyA needs to be degraded after triggering light signaling to prepare for the next round of signaling (i.e., desensitization), so protein degradation might be the important regulation step for phyA signaling. On the other hand, phyB is light-stable and mediate low fluence response (LFR) and R-HIR of plants. The VFR shows photoreversibility, so phyB functions in a typical photoreversible manner between inactive Pr and active Pfr forms. Thus, the function of phyB is regulated by dark reversion (for example, active in the light and inactive in the dark) rather than by proteolysis. Therefore, plant light signaling mediated by phyA and phyB could be regulated by phosphorylation in the NTE via accelerated protein degradation and dark reversion, respectively. However, further studies are necessary to determine exact autophosphorylation sites on phyB, because Ser-86 phosphorylation was only investigated in plants. Regarding to this, it should be noted that another site of phyB phosphorylation has been reported, in which light-induced phosphorylation of Tyr-104 inhibits the binding to PIF3 [85]. Thus, it is possible that phyB is phosphorylated at multiple sites. Overall, there is no doubt about the significant role of phosphorylation for the function of plant phytochromes.

Current data on the phosphorylation of phyA and phyB suggest that the action of both phytochromes can be negatively regulated by phosphorylation. In addition, the phosphorylation of both phyA and phyB affect protein-protein interactions with signaling partners and also provide inactivation mechanisms, i.e., phyA via protein degradation and phyB via dark reversion. The observation that phytochromes are phosphoproteins also suggests the existence of protein kinase(s) that phosphorylate phytochromes and protein phosphatase(s) that dephosphorylate them. However, despite extensive studies of phytochrome-interacting proteins, there is no report thus far of a protein kinase that can phosphorylate phytochromes. On the other hand, a few protein phosphatases have been reported as being able to interact with and dephosphorylate phytochromes, which include FyPP (flower-specific phytochrome-associated protein phosphatase), PAPP5 (phytochrome-associated protein phosphatase 5), and PAPP2C (phytochrome-associated protein phosphatase type 2C) [50,86,87]. All these protein phosphatases act as positive regulators in the phytochrome signaling, suggesting that the phytochrome signaling is regulated by reversible phosphorylation. With current knowledge, it can be suggested that phytochrome phosphorylation decreases signaling flux by reducing the amounts of active phytochromes via either protein degradation or dark reversion, while phytochrome dephosphorylation increases the signaling flux by enhancing phytochrome interaction with signaling partners, as well as either increased protein stability or reduced dark reversion rates.

Considering that there is no report of a protein kinase on phytochromes, it is postulated that phytochromes are autophosphorylated and then dephosphorylated by phytochrome-associated protein phosphatases. Indeed, all the purified recombinant phytochromes of *A. thaliana*, *Pisum sativum* (pea), *A. sativa* (oat), and *Brachypodium distachyon* show autophosphorylation [88]. However, the reported phosphorylation sites cannot be explained by autophosphorylation. Although the phosphorylation sites in the NTE are assumed to be autophosphorylated, phosphorylation sites in the hinge region such as Ser-599 of AsphyA and Ser-590/Thr-593/Ser-602 of AtphyA are not autophosphorylated. Moreover, Tyr-104 phosphorylation of phyB might not be autophosphorylated, because phytochromes are known as serine/threonine kinases [89]. Therefore, it will be necessary to find protein kinase(s) that can phosphorylate phytochromes in the future.

## 5. Protein Kinase Activities of Plant Phytochromes in Plant Light Signaling

Phytochromes exhibit autophosphorylation, although there is no reported protein kinase on them. This suggests the importance of intrinsic protein kinase activity. Moreover, the first discovered cyanobacterial phytochrome Cph1 shows autophosphorylation and histidine kinase activity [90]. Based on these facts, there were a lot of debates on whether plant phytochromes are protein kinases or not. To this end, it was shown that purified recombinant plant phytochromes exhibit a serine/threonine kinase activity, proposing that eukaryotic phytochromes are histidine kinase paralogs with serine/threonine specificity [89]. Subsequently, several proteins were reported to be phosphorylated by phytochromes in vitro, such as histone H1, PKS1, cryptochromes, and Aux/IAA proteins [49,91,92,93]. Later, the Pfr form of phytochromes has shown to induce rapid phosphorylation of PIFs preceding degradation in plants, suggesting the possible role of the phytochrome kinase activity for photomorphogenic development [66,67,68]. After these discoveries, it has been established that the function of PIFs is regulated by phosphorylation and phytochromes are necessary for the phosphorylation of PIFs [14]. However, at that time, it was not clear whether phytochromes phosphorylate PIFs directly or not. Recently, AsphyA mutants displaying reduced kinase activities were obtained and further analysis confirmed that their transgenic plants exhibit hyposensitive responses to FR light [88]. Moreover, both type I and type II phytochromes strongly phosphorylate PIF3 in vitro, and FR light-induced phosphorylation and protein degradation of PIF3 are significantly prevented in the transgenic plants. Collectively, these data demonstrate a positive relationship between the kinase activity of phytochromes and photomorphogenic responses in plants. Therefore, it is now evident that plant phytochromes are protein kinases and that the kinase activity is important for the function in plants.

During in vitro and in vivo phosphorylation experiments of PIF3 by phytochromes, the differences in the phosphorylation levels of PIF3 have been observed with slow-migrating proteins due to multiple phosphorylations, i.e., hyperphosphorylation of PIFs in plants. Thus, it is suggested that phytochromes alone are not enough to mediate the hyperphosphorylation of PIF3 in plants, although phytochromes are able to initiate PIF3 phosphorylation. This means that additional protein kinase(s) may be necessary to induce the multiple phosphorylations of PIF3 in plants. Indeed, after the report of phytochromes as protein kinases that can phosphorylate PIF3 [88], at least two more types of protein kinases have been reported for the phosphorylation of PIFs in plants: BIN2 (brassinosteroid insensitive 2) and PPKs (photoregulatory protein kinases; PPK1 to PPK4) [94,95]. Therefore, it is likely that PIFs are regulated via phosphorylation by multiple protein kinases in plants. However, it should be noted that phytochromes are the most important kinases because the phosphorylation of PIFs is not observed in the absence of phytochromes.

If we consider phytochromes as protein kinases, the next question would be substrate proteins other than PIFs. Among the reported proteins that can be phosphorylated by phytochromes, histone H1 and IAA/Aux proteins are known to be phosphorylated in vitro only and PKS1 is localized in the cytoplasm, so the phosphorylation of these proteins by phytochromes might not be important for the plant light signaling. In case of cryptochromes, i.e., blue-light photoreceptors in plants, recent studies propose a mechanism of phytochrome-cryptochrome coaction with new genes, named as BICs (blue-light inhibitor of cryptochromes) [96,97]. Since it has been shown that phyA regulates blue-light signaling in plants [54], as well as far-red and red light signaling, it will be interesting to investigate phytochrome-mediated blue light signaling via the phosphorylation of cryptochromes by phytochromes. More interestingly, it has been known that FHY1/FHL shows rapid R light-induced phosphorylation in a phyA-dependent manner [98,99]. More recently, it was shown that phosphorylation of serine residues near the NLS prevents FHY1 binding to IMPα [44]. Previously, the dissociation rate of phyA-FHY1/FHL complex is a principal determinant for the accumulation of phyA in the nucleus under FR light, i.e., phyA associates with FHY1/FHL in the cytoplasm but the phyA-FHY1/FHL complex should be dissociated in the nucleus for the phyA function [8]. Collectively, it can be postulated that the phyA-FHY1/FHL complex might be dissociated via the phosphorylation of FHY1/FHL in the nucleus, which makes phyA to function in FR light signaling. However, the protein kinase(s) that can phosphorylate FHY1/FHL is not known, so it is worthwhile to investigate the possibility of FHY1/FHL phosphorylation by phyA. Another interesting report is about the phosphorylation of COP1. A recent study reported that a serine/threonine kinase PINOID (PID) phosphorylates COP1 at Ser-20, which represses COP1 activity and promotes photomorphogenesis [100]. It has been shown that the direct interaction of photoactivated photoreceptors with the COP1-SPA complexes represses its activity via disruption of the COP1-SPA interaction and nuclear exclusion of COP1 [70]. These data suggest that COP1 phosphorylation might be necessary for the dissociation of the COP1-SPA complexes and also for the nuclear exclusion of COP1. Since the mechanistic link between photoreceptor signaling and COP1 phosphorylation remains to be identified, it might be worthwhile to investigate whether COP1 and/or SPA proteins can be phosphorylated by phytochromes in the future.

**Figure 3:**
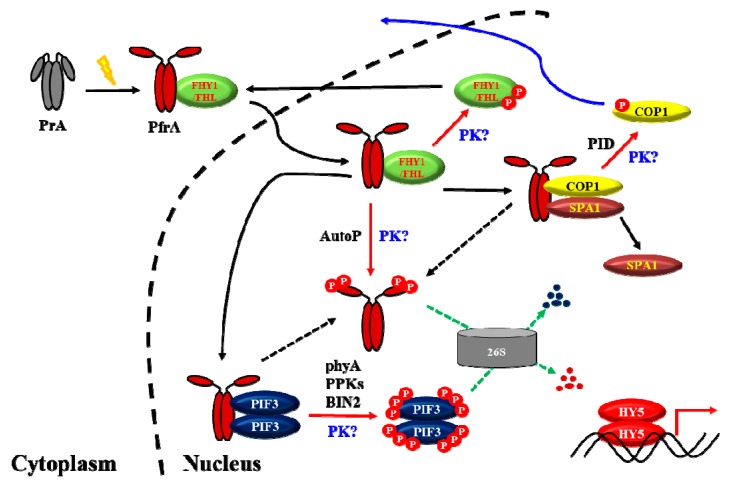
A proposed model to explain the molecular mechanisms for phyA signaling under a light condition. PrA, the Pr form of phyA; PfrA, the Pfr form of phyA; 26S, the 26S proteasome complex; P, phosphate; PK, unknown protein kinase(s). As a protein kinase, PfrA is autophosphorylated (AutoP) and phosphorylates substrate proteins such as PIF3. So far, PIF3 is also known to be phosphorylated by PPKs and BIN2, and COP1 is phosphorylated by PID. However, the protein kinase(s) that can phosphorylate phytochromes are unknown (labeled as “PK?”). In addition, it is not known how FHY1 and FHL can be phosphorylated (i.e., another label with “PK?”). Moreover, it is possible that PIF3 and COP1 might be phosphorylated further by unknown protein kinase(s). As shown in this model, phosphorylation (shown as red arrows) takes significant portions in the phytochrome signaling in plants. In summary, the function of phytochromes is to inactivate the negative regulators for photomorphogenesis such as PIF3 and COP1-SPA1 complex, which eventually express and stabilize HY5 to initiate photomorphogenic development. At the same time, phosphorylated PfrP is rapidly degraded via the ubiquitin 26S proteasome pathway (shown as green arrows) for an efficient desensitization of the phyA signal, which is necessary for responses to subsequent changes in fluctuating light environments.

With the above advances, a possible model for the function of phytochromes as protein kinases can be speculated (Figure 3). For simplicity, we use phyA for the model. Once photoactivated, the Pr form of phyA (PrA) is transformed into the Pfr form (PfrA) that can interact with NLS/NES sequence-containing FHY1/FHL proteins. The PfrA-FHY1/FHL complex is then localized into the nucleus with the help of IMPα. In the nucleus, FHY1/FHL proteins are phosphorylated by unknown protein kinase(s), which releases PfrA from the complex. Thus, light is necessary for the translocation of phytochromes into the nucleus. In addition, the kinase activity of phytochromes could be stimulated in the nucleus, because the kinase activity is known to be stimulated in the presence of polycations such as histones [88,89,93]. Once localized in the nucleus, PfrA directly phosphorylates PIF3 whose hyperphosphorylation can be achieved with other protein kinases such as PPKs, BIN2, and possibly unknown protein kinase(s), inducing PIF3 degradation via the ubiquitin 26S proteasome proteolytic pathway. At the same time, PfrA interacts with the COP1-SPA complex and COP1 is phosphorylated by PID and/or unknown protein kinase(s), which may induce the dissociation of the complex and the nuclear exclusion of COP1. After transducing light signals, PfrA is phosphorylated by itself (i.e., autophosphorylation; AutoP) and/or by unknown protein kinase(s), which induces protein degradation via the ubiquitin 26S proteasome pathway. As mentioned before, the inactivation of negative regulators such as PIFs and COP1-SPA complexes contributes to the initiation of photomorphogenesis, and the degradation of phyA under light conditions plays a role for desensitization. When plants are under darkness or weak light conditions, PrA is biosynthesized again for sensing the next light signals. Although it is not shown in this model, it is also notable that HY5 is reported to be phosphorylated by yet unknown protein kinase(s), which lowers the binding affinity to COP1 and also influences DNA binding [101,102]. Thus, the transcription activity and protein stability of HY5 might be regulated by phosphorylation.

In the case of phyB, the facilitator(s) for importing into the nucleus is not elucidated. In addition, autophosphorylation might accelerate dark reversion, rather than protein degradation as shown in phyA, which plays a role in signal desensitization. However, phyB also phosphorylates PIFs for protein degradation and induces the dissociation of the COP1-SPA complexes. Thus, phyB mediates photomorphogenic development in a similar way to phyA, i.e., by inactivating the negative regulators for photomorphogenesis.

## 6. Conclusions and Perspectives

It is evident that reversible phosphorylation plays important roles in the regulation of phytochrome signaling and that plant phytochromes are autophosphorylating protein kinases. However, phosphorylation sites and the effects of phosphorylation are different between phyA and phyB. The phosphorylation sites of phyB including autophosphorylation remain to be identified. In particular, it is not clear whether the hinge region of phyB is phosphorylated or not, although the autophosphorylation sites might reside in the NTE. Autophosphorylation of phyA and phyB increases the rates of protein degradation and dark reversion, respectively, while phosphorylation of phyA in the hinge region and that of phyB at Tyr-104 affect the interaction with downstream signaling partners. Considering the phosphorylation of phyA in the hinge region and phyB at Tyr-104, there should be protein kinase(s) that can phosphorylate phytochromes. Over the decades, extensive research was performed to identify phytochrome-interacting protein kinases, but there is no report so far. Therefore, it is necessary to isolate protein kinase(s) that act on phytochromes in the future.

The fact that phytochromes are protein kinases is important to understand why they have many interacting proteins in plants [45]. If a protein has an enzymatic activity, the protein might have many substrates. Thus, as enzymes with a kinase activity, phytochromes may interact with lots of proteins as substrates. In *A. thaliana*, eight PIFs are known to physically interact with phytochromes. Thus, it will be interesting to investigate the phosphorylation of these PIFs by different phytochrome species. In addition, besides PIFs, it will be necessary to find other substrate proteins that can be phosphorylated by phytochromes. According to the recent advances, there are several candidates for the substrate proteins, such as cryptochromes, FHY1/FHL, and COP1-SPA complexes. Therefore, the phosphorylation of these proteins by phytochromes and its functional roles in plant light signaling would be interesting topics in the future. 

Recently, the top twenty Arabidopsis genes of all time were acknowledged in TAIR (The Arabidopsis Information Resource). In the list, phyB and phyA are ranked as the 1st and 4th, so phytochromes are one of the most studied genes so far. Especially, with the problems of global warming, plant scientists are interested in the study of thermomorphogenesis, i.e., morphological changes that are likely to contribute to adaptive growth acclimation to usually elevated ambient temperatures. Regarding this, phyB has been reported as a thermosensor in plants [9,10]. Moreover, it is known that PIF4 is the master regulator of thermomorphogenesis [103]. Therefore, the regulation of PIF4 via phosphorylation by phyB would be another interesting topic for the future.
